# Factors influencing disaster preparedness behaviors of older adults

**DOI:** 10.1371/journal.pone.0315617

**Published:** 2025-02-28

**Authors:** Kai-Min Liao, Yih-Jin Hu

**Affiliations:** 1 Department of Health Promotion and Health Education, National Taiwan Normal University, Taipei, Taiwan; 2 Policy and Socio-Economics Division, National Science and Technology Center for Disaster Reduction, New Taipei, Taiwan; International University of Business Agriculture and Technology, BANGLADESH

## Abstract

This study examines the heterogeneity in disaster preparedness behaviors among older adults and the factors that influence them, with the aim of offering policy recommendations to mitigate casualties among older adults during natural disasters. This is a secondary data analysis of cross-sectional data involving 394 participants aged 65 and above, with data sourced from the seventh wave of the Basic Social Change Survey conducted by Academia Sinica. These cross-sectional data were collected through face-to-face interviews, where interviewers conducted one-on-one questioning to gather general information and assess disaster preparedness. Hierarchical regression analysis was employed to explore the relationship between various factors and disaster preparedness behaviors. Descriptive statistics show that among the six disaster preparedness behaviors, 32.5% of the elderly moved vehicles or household items to a safe location, and 27.2% secured cabinets or large appliances. The remaining four disaster preparedness behaviors—including purchasing disaster insurance, preparing a disaster emergency kit, identifying and planning evacuation locations and routes, and participating in disaster response drills—were exhibited by less than 11.9% of the participants. Hierarchical regression showed that younger age, higher education, lower income, better health, community involvement, disaster experience, and higher perceived risk were associated with increased preparedness among older adults. The study found that most older adults do not invest time or money in disaster preparedness. Government agencies should encourage older adults to participate and account for their heterogeneity, such as through targeted interventions in health promotion, disaster response education, and social support. Initiatives like health check-ups, exercise classes to improve physical fitness, and simple, understandable disaster response courses can enhance risk perception. For high-income groups, emphasizing the importance of disaster preparedness through data and real-life examples is crucial. Older adults should also be encouraged to join community organizations and disaster drills, and a platform for sharing disaster experiences should be established to improve overall disaster resilience.

## Introduction

Older adults, due to age-related physical decline, chronic health conditions, mobility limitations, and social isolation, are considered a high-risk group during disasters. Their increased vulnerability, particularly in response and evacuation scenarios, hinders their ability to react quickly and protect themselves [1]. Furthermore, the Sendai Framework for Disaster Risk Reduction highlights the need to prioritize the protection of vulnerable groups, especially the elderly, as a key element of disaster risk reduction strategies. The framework emphasizes the importance of addressing their specific needs to ensure adequate support and resources before, during, and after disasters [2]. According to the Taiwan Ministry of the Interior, Taiwan entered the stage of an aging society in 2018, which means the percentage of older adults surpassed the threshold of 14% and is expected to exceed 20% by 2025, meeting the criteria for a “super-aged society.” [3] Previous studies have indicated that older adults are one of the most vulnerable groups in disasters [4,5]. For example, during the Kobe earthquake in 1995, about 57% of the deceased were elderly, a proportion significantly higher than their representation in the population structure. Similarly, during Hurricane Katrina in 2005, approximately 67% of the deceased were elderly, despite this age group only accounting for 12% of the total population [6]. Additionally, in the 2011 Great East Japan Earthquake, over 15,000 people perished, with 66.1% of them being aged 60 and above [7]. According to the mortality data from natural disasters provided by the Ministry of the Interior between 2014 and 2017, the fatality rate for older adults was 0.81, indicating that Taiwan’s older adult population is disproportionately affected by disasters. This highlights the insufficient attention given to their needs and voices in disaster awareness and preparedness. As natural disasters become more frequent and severe, and Taiwan’s population continues to age rapidly, it is essential to implement more comprehensive and effective measures to enhance the disaster resilience of older adults.

As age increases, the deterioration of physical and mental health, reduced economic resources, and a potential decrease in social network support may occur [8]. Economic resources play a crucial role in disaster preparedness and response. The most common reason for the increased vulnerability of older adults in disasters is the deterioration of health conditions, with the likelihood of having chronic health conditions or special needs increasing with age. This poses challenges for older adults in receiving disaster warnings and taking protective measures, such as finding shelter during earthquakes. Those with disabilities or health issues may find it more difficult to prepare for evacuation, evacuate without assistance, or participate in post-disaster cleanup [9]. Due to physical decline and chronic illnesses, older adults face obstacles in daily life and may have difficulty perceiving disaster signs or identifying warnings during disasters. For instance, poor night vision and peripheral vision in older adults can lead to difficulties in unfamiliar environments or during rapid evacuations. Moreover, they may be unaware of evacuation directions, have slower walking speeds due to physical decline, or require assistive tools like canes, walkers, or wheelchairs, making it challenging for them to react promptly and evacuate in the event of an earthquake [10]. Researchers found that individuals in poorer health, those with disabilities, or those suffering from multiple chronic diseases are less likely to develop a clear emergency evacuation plan compared to those in better health [11].

Older adults with lower economic status are more likely to report having insufficient insurance coverage and fewer opportunities to receive federal assistance in disasters [5]. Research on older Americans by Durant found that although the poverty rate among older adults decreased and was lower than that of non-elderly adults, many older adults relied solely on fixed incomes, limiting their ability to mobilize and access resources in emergencies [12]. Bolin and Klenow observed that older adults faced more challenges in obtaining post-disaster loans compared to younger individuals and had greater difficulty bridging the gap between financial losses and insurance coverage. Therefore, older adults were almost twice as likely as younger individuals to experience a decline in their standard of living after a disaster [13].

Social capital, as explained by Bourdieu, involves resources that can be accessed through social networks [14]. Social capital is crucial at all stages of natural disasters, including warning and information dissemination through social networks, as well as assistance from family and friends in pre-disaster preparedness activities [15]. Due to declines in physical and cognitive abilities, older adults rely more on the assistance of others. However, the size of their social networks typically decreases with age [16]. Older adults are less likely to seek help from family, neighbors, or friends during or after a disaster, and they are also less likely to receive formal assistance from government and community organizations [17]. Klinenberg emphasized the importance of social network relationships for survival during the 1995 Chicago heatwave, noting that disaster-related deaths were predominantly among isolated older adults [18].

Past experiences may lead older adults to prepare for disasters; those severely affected in past disasters know how difficult and slow it is to receive emergency assistance [19]. Therefore, they tend to be better prepared for disasters. On the other hand, older adults who have experienced minor impacts or no effects in disasters usually have insufficient preparation for possible future disasters, exposing them to higher risks of illness and death. Compared to younger people, older adults may prioritize disaster preparedness due to past experiences or concerns about the future [11], but some literature also points out that older adults may lack sufficient financial resources to purchase items needed for disaster preparedness, such as emergency food, water, medications, battery-powered radios, or televisions [19].

Risk perception pertains to individuals’ comprehension or assessment of particular hazards that could result in harm to life or property. These perceptions, whether at the individual or community scale, constitute a crucial social element of managing disaster risks. They influence reactions to alerts and enhance community preparedness initiatives [20]. Many studies have explored the relationship between age and risk perception [21, 22]. One notable study identified a significant connection, revealing that older adults tend to have a heightened sense of risk perception [23].

Most disaster management personnel recognize the importance of self-efficacy in disaster preparedness. The Sendai Framework for Disaster Risk Reduction 2015-2030, adopted at the Third World Conference on Disaster Risk Reduction, echoes this view, emphasizing the importance of enabling people to learn from disaster experiences and prepare in advance for potential future disasters [2]. If the public actively engages in disaster preparedness, combined with the government’s structural disaster mitigation efforts, the risk of disaster impact on the public can be reduced [24]. With the acceleration of population aging, improving self-efficacy among older adults to reduce their risk of natural disasters is becoming increasingly important. Between 2005 and 2023, 85.09% of the villages that received landslide warnings had over 14% of their population comprised of older adults. This indicates the importance of disaster preparedness efforts for older adults, including disaster prevention education, planning evacuation routes, and participation in disaster response drills. The purpose of this study is to understand the relationship between the characteristics of older adults and their disaster preparedness behaviors. Based on the findings, more targeted policy recommendations will be proposed for disaster management agencies.

## Materials and methods

### Population and data collection

This study used data from the seventh wave of the Basic Social Change Survey conducted by the Academia Sinica, comprising 1,933 samples. Household registration data files served as the sampling frame, and the sampling design followed a stratified multi-stage probability proportional to size (PPS) method, involving geographic stratification, township/city district, village/neighborhood [25]. Since this study focuses on older adults, we selected participants aged 65 and above from the 1,933 samples as a subsample for further analysis.

### Measurement

For this study, we focused on individuals aged 65 and above, resulting in a sample of 394 participants. However, since the survey did not cover medical institutions, some older adults residing in such facilities are not included in the scope of this study. After applying unequal probabilities of selection and sampling weights, the weighted sample size became 398. We utilized an F-test with multiple linear regression in a fixed model to analyze the increase in R^2^ post hoc, with an effect size of f^2^ set at 0.15 and a p-value of 0.05. Our total sample size exceeded the minimum required sample size of 146, ensuring adequacy for the analysis.

### Outcome variable: Disaster preparedness

This study used the questionnaire set “Have you done the following disaster preparedness work?” to calculate the actual disaster preparedness behaviors engaged by the respondents, as the outcome variable “disaster preparedness behavior score.” The disaster preparedness behaviors consist of six items: 1. Moving vehicles or household items to a safe location; 2. Purchasing disaster insurance; 3. Repairing cabinets or large household appliances; 4. Preparing a disaster emergency kit; 5. Identifying and planning evacuation locations and routes; 6. Participating in disaster response drills. If none of the actions were taken, the score is 0. The outcome variable was a summative interval scale variable adding the total score of the six items, ranging from 0 to 6. Higher scores indicate higher levels of public disaster preparedness.

### Antecedent variable: Physical condition and mobility impaired

Respondents were asked, overall, do you think your physical condition is good or not? From 1 very good to 5 very bad. The Respondents were also asked if they are mobility impaired. If yes, the code is 1; if not, the code is 0.

### Antecedent variables: Socio-demographic characteristics

The demographic characteristics include gender (male and female, coded as 0 and 1, respectively), age (reported as the actual age), educational level (classified into 5 levels: illiterate, elementary school, middle school, high school, and college, coded as 1 to 5), average monthly income (divided into 8 levels: no income, below NTD 10,000, NTD 10,000–20,000, NTD 20,000–30,000, NTD 30,000–40,000, NTD 40,000–50,000, NTD 50,000–60,000, and over NTD 60,000, with an exchange rate of approximately NTD 32 =  USD 1, coded as 1 to 8), and area of residence (urban and rural, coded as 0 and 1). This study assumed socio-demographic characteristics were related to economic resources as mentioned in the literature review.

### Antecedent variable: Social network

Respondents were asked to report their affiliations with different organizations, including the following: (1) political associations; (2) community management committees; (3) social service organizations; (4) religious organizations; (5) recreational organizations; (6) worker unions; and (7) others. If respondents participated in any of the above, the code is 1; if they did not participate in any, the code is 0.

### Antecedent variable: Disaster experiences

This was a summarized variable measured by three questions: “How many times have you experienced a flood caused by typhoons or heavy rains?“, “How many times have you experienced geohazards caused by typhoons or heavy rains, such as landslides and debris flows?“, and “How many times have you experienced earthquakes?“. If respondents experienced any of the above disasters, the code is 1; if they did not experience any, the code is 0.

### Antecedent variable: Risk perception

The questions include “Do you usually worry about yourself or your family being affected by typhoons or floods?” and “Do you usually worry about yourself or your family being affected by earthquakes?”, with the level of concern ranging from 1 (not worried at all) to 5 (very worried).

### Data analysis

The IBM SPSS statistics 23.0 software program (IBM Corp., Armonk, New York, USA) was used to analyze the data quantitatively. This study used hierarchical multiple regression to examine the factors predicting disaster preparedness. Based on the literature, the predictor variables were categorized into four levels: physical condition and mobility impairment, sociodemographic characteristics, social networks, and natural disaster-related variables (disaster experience, risk perception). Regression analysis was conducted sequentially, adding one variable at a time.

### Ethical approval

The data analyzed in this study has been approved by the Research Ethics Committee for Humanities and Social Sciences at Academia Sinica (AS-IRB-HS07-108107). The data collection was conducted through one-on-one interviews. The survey started on 30/06/2019 and ended on 31/12/2019. It targeted individuals aged 18 and above (born on or before 31/12/2019) with household registration in Taiwan, excluding Kinmen County and Lienchiang County in Fujian Province. Interviewers obtained participants’ consent verbally; if a participant did not consent, the interview was terminated, and the reason for refusal was recorded in the survey log.

## Result

### Descriptive statistics

Descriptive statistics of each variable are shown in Table 1. The average age of respondents was 73.15 years old, with slightly more females than males (49.70%). The mode of educational attainment is primary school, accounting for 45.5%. The majority were non-disabled (81.80%), with nearly half having a monthly income below ten thousand yuan. Approximately 80% of the elderly population resides in urban areas (76.57%). Self-perceived physical health (3.62) was above the midpoint, and the percentage of organization participants (53.80%) was slightly higher than non-participants. About two third of respondents had no disaster experience, and their perceived risk of natural disasters was slightly higher than the midpoint (2.79, the lower the score, the higher the risk perception).

**Table 1:** Respondent characteristics description statistics (n = 398).

Variables	Categories	% or M(SD)
Age (year)		73.15(6.61)
Gender	Male	49.75%
	Female	50.25%
Education level	Illiterate	17.77%
	Elementary school	45.48%
	Middle school	12.72%
	High school	11.79%
	College	12.24%
Mobility impaired	Yes	18.00%
	No	82.00%
Average monthly income	No income	7.76%
	Below NTD10,000	44.35%
	NTD 10,000–20,000	21.30%
	NTD 20,000–30,000	9.48%
	NTD 30,000–40,000	6.68%
	NTD 40,000–50,000	3.53%
	NTD 50,000–60,000	2.94%
	Over NTD 60,000	3.96%
Physical condition		3.62(1.13)
Participating organizations	Yes	53.77%
	No	46.24%
Disaster experience	Yes	35.40%
	No	64.60%
Risk perception		2.79(1.31)
Area of residence	Urban	76.57%
	Rural	23.43%

Abbreviations: M = mean; SD = standard deviation.

The descriptive results of the survey on the disaster preparedness behaviors of older adults were shown in Table 2; among the six disaster preparedness behaviors, the most common one was moving cars or household items to a safe place (32.50%), and the least common one was participating in disaster response drills (4.37%). However, none of the disaster preparedness behaviors were performed by more than 50% of the respondents, indicating that the disaster preparedness behaviors of older adults were generally lacking.

**Table 2:** Elderly disaster preparedness behavior description statistics (n = 398).

Disaster preparedness	Frequency		Percentage	
	No	Yes	No	Yes
Move vehicles or household items to a safe location	269	129	67.60%	32.50%
Purchase disaster insurance	357	41	89.63%	10.37%
Fix the cabinets or large appliances at home	291	107	73.12%	26.88%
Prepare a disaster preparedness kit	352	46	88.32%	11.68%
Understand and plan evacuation locations and routes	353	45	88.71%	11.29%
Participate in disaster response drills	381	17	95.63%	4.37%

### Factors predicting disaster preparedness behavior

This study conducted four-stage hierarchical regression analysis to determine the significant influencing factors associated with disaster preparedness behavior among older adults (Table 3). Given the pronounced disparities between older adults and other socio-demographic groups, particularly in terms of health conditions, the first stage incorporated physical condition and mobility impaired. The second stage included socio-demographic variables, the third stage encompassed social network conditions, and the final stage featured variables commonly employed in disaster preparedness research, specifically disaster experience and risk perception. The results satisfied the linearity and normality assumptions in the linear regression analysis. The Durbin-Watson statistic was 1.82, which indicated that there were not autocorrelations. The tolerance was 0-1, and VIF’s were 1.04-1.77, below 10, so there was no multicollinearity.

**Table 3:** Factors influencing natural disaster preparedness(n = 398).

Variables	b	*β*	*t(p)*	b	*β*	*t(p)*	b	*β*	*t(p)*	b	*β*	*t(p)*
(contrast)	1.735			3.667			3.495			3.621		
Physical condition	−0.195	−0.188	−3.608^***^	−0.134	−0.129	−2.452^*^	−0.124	−0.120	−2.291^*^	−0.150	−0.144	−2.842^***^
Mobility impaired	−0.187	−0.061	−1.170	−0.128	−0.042	−0.819	−0.086	−0.028	−0.551	−0.077	−0.025	−0.513
Gender (ref = male)				−0.105	−0.044	−0.849	−0.106	−0.045	−0.868	−0.190	−0.080	−1.590
Age				−0.028	−0.153	−2.912^**^	−0.023	−0.128	−2.416^*^	−0.017	−0.097	−1.881
Education level				0.123	0.157	2.411^*^	0.113	0.144	2.226^*^	0.140	0.179	2.847^**^
Average monthly income				−0.072	−0.153	−2.579^*^	−0.073	−0.154	−2.618^**^	−0.066	−0.140	−2.452^*^
Area of residence (ref = rural)				−0.375	−0.135	−2.649^**^	−0.363	−0.130	−2.586^*^	−0.367	−0.132	−2.683^**^
Participating in organizations (ref = no)							0.335	0.141	2.763^**^	0.290	0.122	2.451^*^
Disaster experience (ref = no)										0.261	0.106	2.147^*^
Risk perception										−0.191	−0.210	−4.124^***^
*R* ^*2*^	.044			.123			.142			.202		
*adj. R* ^*2*^	.039			.106			.122			.179		
*ΔR* ^*2*^				.067			.016			.057		
*F(p)*	8.375^***^			7.230^***^			7.397^**^			9.021^***^		

*: *p* < .05; **: *p* < .01; ***: *p* < .001.

Model 1 of the regression included physical condition and mobility impaired, which was statistically significant (F = 8.375, *p* < .001) and explained about 3.9% of the variance. The worse the physical condition, the fewer disaster preparedness behaviors among older adults (β = −.188, *p* < .001).

Model 2 of the regression included physical condition, mobility impaired, and socio-demographic characteristics, which was statistically significant (F = 7.230, *p* < .001) and explained about 10.6% of the variance, 6.7% higher than Model 1. As age increased (β = −.153, *p* < .01), education level decreased (β =.157, *p* < .05), average monthly income increased (β = −.153, *p* < .05), and older adults who live in rural area (β = −.135, *p* < .01), less preparedness behavior was evident among older adults.

Model 3 of the regression included physical condition, mobility impaired, socio-demographic characteristics, and participation in organization (reference=no). This model was significant (F =  7.397, p < .01) and explained 12.2% of the variance, which represents an increase of 1.6% compared to Model 2. Older adults who participate in organizations exhibit more disaster preparedness behaviors (β = .141, *p* < .01).

Finally, physical condition, mobility impaired, socio-demographic characteristics, participation in organization, and the experience and awareness factors of natural disasters were input into model 4, and it was found to be statistically significant (F =  9.021, *p* < .001). The percentage of explained variance in the end was 17.9%, which represents an increase of 5.7% compared to Model 3. The higher the experience of disaster (β =.106, *p* < .05) and the higher the risk perception (β = -.210, *p* < .001), the significantly increased disaster preparedness behavior.

## Discussion

We analyzed the study results by comparing them with existing academic literature. The finding that older adults exhibit lower levels of disaster preparedness aligns with studies conducted in other countries [11,26]. This study identifies factors influencing disaster preparedness behaviors among older adults, and the results not only enrich the existing literature but also highlight the heterogeneity within this demographic group.

As shown in Table 3, all four models indicate a decline in disaster preparedness behaviors associated with several factors: deteriorating physical health, increasing age, decreasing education level, rising average monthly income, rural residency, organizational participation, and heightened disaster experience and risk perception. These findings have important implications for disaster management policy and practice.

Physical health is a significant factor. Older adults with poorer health are less likely to engage in disaster preparedness, consistent with previous studies [1,9,27], suggesting that limited mobility may reduce their motivation. This underscores the need for special support and resources for older adults with mobility challenges.

Age also affects preparedness. Our findings show a decline in preparedness as age increases, which is supported by several studies [11,28,29]. This trend may be due o diminished physical capabilities, which can hinder participation in preparedness activities [1,9,27], as well as greater reliance on family members, reducing personal preparedness [30]. Tailored strategies for different age groups, particularly the oldest, are crucial. For high-aged elderly, providing personalized support such as home visits for preparedness assessments and easy-to-understand educational materials can significantly enhance their disaster preparedness.

Education level plays a clear role in influencing preparedness. Higher levels of education are associated with more proactive disaster preparedness, consistent with prior studies [19,27]. Increasing disaster education and raising risk awareness among older adults could be effective in boosting overall preparedness.

Income presents a somewhat surprising relationship with preparedness. Contrary to previous research [12], we found that older adults with higher incomes were less likely to engage in preparedness activities. This may be because higher-income individuals live in safer environments and perceive lower levels of risk [31], or because financial security lessens concerns about potential losses [32]. The results highlight the importance of customized approaches for different income groups. For high-income older adults, strategies should emphasize the importance of property protection (such as disaster insurance) and highlight that even in safer environments, risk-reducing measures remain essential.

Area of residence also contributes to variations in preparedness. Older adults in urban areas are more likely to engage in disaster preparedness than their rural counterparts, likely due to differences in access to resources and risk perception [33]. This highlights the need to improve disaster education and resource allocation in rural areas.

Organizational participation is another factor linked to preparedness. Older adults who participate in organizational activities tend to exhibit more proactive preparedness behaviors, aligning with social capital theory [17,27]. This underscores the importance of social networks in promoting preparedness and provides a strong case for community-based programs.

Finally, disaster experience and risk perception strongly influence preparedness. Older adults with disaster experience and higher risk perception are more likely to engage in preparedness, consistent with several studies [11,20–23]. This points to the value of incorporating real-life experiences and risk assessments into disaster education.

These findings reveal the complexity and diversity of disaster preparedness behaviors among older adults. Policymakers and practitioners must adopt multifaceted approaches, tailoring strategies to account for differences in health, education, and income, while also strengthening community involvement and risk awareness. Future research should explore how these factors interact and how insights can be translated into effective interventions.

### Limitations of the Study

This study is not without limitations. First, the preparedness behaviors were self-reported by participants, which may have been influenced by social desirability bias. Second, as a cross-sectional analysis, causal inferences cannot be drawn from these data. Future longitudinal studies could further clarify the relationships between the variables. Third, the measurement of disaster preparedness behaviors through a summative scale might not capture the varying importance of different behaviors. In future studies, we will also consider analyzing each disaster preparedness behavior separately and then attempt to formulate more refined scales to more accurately assess the importance of different disaster preparedness behaviors. Finally, the sample from Taiwan may not be fully applicable to other cultural or geographical contexts. Future research could consider expanding the sample to cover more cultural and geographical areas to enhance the generalizability of the findings.

## Conclusion

This study contributes to existing literature by focusing on the heterogeneity among older adults and identifying the factors that influence their disaster preparedness behaviors. The findings reveal that a high proportion of older adults do not invest time or resources into disaster preparedness, despite being the most vulnerable to the impacts of disasters.

It is recommended that government interventions address the factors identified in the study through the following strategies

### Health Promotion and Physical Well-being

Governments should establish more health promotion activities for older adults, such as regular health check-ups, exercise classes, and nutrition workshops. These initiatives not only improve their physical condition but also enhance their ability to respond to disasters and reduce the physical burden during such events.

### Disaster Education and Knowledge Dissemination

For older adults with lower levels of education, governments can develop simple and accessible disaster preparedness courses. These can be offered through community centers, senior academies, and online platforms to ensure that older adults learn essential self-rescue and emergency measures, strengthening their risk awareness and preparedness.

### Targeted Risk Awareness Education

High-income older adults may underestimate the importance of disaster preparedness, believing financial resources are sufficient to cope with the aftermath. Governments should provide personalized disaster education for these groups, using data and examples to highlight the severity of potential disasters and emphasize that preparedness requires concrete actions beyond financial resources.

### Community Engagement and Social Support Networks

Governments should encourage older adults to participate more in community organizations and disaster drills. Strengthening neighborhood networks can promote mutual aid, and regular simulations can boost their confidence and ability to respond. Establishing “Disaster Response Volunteer Teams,” led by experienced seniors, can also increase participation and preparedness.

### Personalized Risk Perception Education

For older adults with lower risk perception, governments should use simple videos and images to depict the potential impacts of disasters and share real-life examples. Community outreach, such as door-to-door visits, can further raise awareness and disaster preparedness.

### Experience Sharing and Mutual Support Networks

Governments can promote experience sharing among older adults by creating platforms for disaster preparedness discussions or organizing regular sharing sessions. This enables seniors to learn strategies from each other, fostering a greater focus on disaster preparedness.

### Implement disaster preparedness plans tailored to rural environments

The government can promote disaster education and preparedness plans suited to rural areas, integrating local industries and daily living needs. By providing targeted guidance, elderly residents can be effectively engaged in disaster preparedness efforts.
